# Simultaneous determination of 2-(3-hydroxy-5-phosphonooxymethyl-2-methyl-4-pyridyl)-1,3-thiazolidine-4-carboxylic acid and main plasma aminothiols by HPLC–UV based method

**DOI:** 10.1038/s41598-023-36548-9

**Published:** 2023-06-07

**Authors:** Justyna Piechocka, Monika Wyszczelska-Rokiel, Rafał Głowacki

**Affiliations:** Department of Environmental Chemistry, Faculty of Chemistry, University of Lodz, 163/165 Pomorska Str., 90-236 Łódź, Poland

**Keywords:** Bioanalytical chemistry, Medical and clinical diagnostics, Screening

## Abstract

The report presents the first method for simultaneous determination of plasma 2-(3-hydroxy-5-phosphonooxymethyl-2-methyl-4-pyridyl)-1,3-thiazolidine-4-carboxylic acid (HPPTCA), an adduct of cysteine (Cys) and active form of vitamin B6 pyridoxal 5′-phosphate (PLP), as well as total low molecular-weight thiols content, including Cys, homocysteine (Hcy), cysteinyl-glycine (Cys-Gly), and glutathione (GSH). The assay is based on high performance liquid chromatography coupled with ultraviolet detection (HPLC–UV) and involves disulfides reduction with *tris*(2-carboxyethyl)phosphine (TCEP), derivatization with 2-chloro-1-methylquinolinium tetrafluoroborate (CMQT) followed by sample deproteinization with perchloric acid (PCA). The chromatographic separation of obtained stable UV-absorbing derivatives is achieved on ZORBAX SB-C18 (150 × 4.6 mm, 5.0 µm) column using gradient elution with eluent consisted of 0.1 mol/L trichloroacetic acid (TCA), pH 1.7 and acetonitrile (ACN), delivered at a flow rate 1 mL/min. Under these conditions, the analytes are separated within 14 min at room temperature, and quantified by monitoring at 355 nm. Regarding HPPTCA, the assay linearity was demonstrated within a 1–100 µmol/L in plasma and the lowest concentration on the calibration curve was recognized as the limit of quantification (LOQ). The accuracy ranged from 92.74 to 105.57% and 95.43 to 115.73%, while precision varied from 2.48 to 6.99% and 0.84 to 6.98% for intra- and inter-day measurements, respectively. The utility of the assay was proved by application to plasma samples delivered by apparently healthy donors (n = 18) in which the HPPTCA concentration ranged from 19.2 to 65.6 µmol/L. The HPLC–UV assay provides complementary tool for routine clinical analysis, facilitating further studies on the role of aminothiols and HPPTCA in living systems.

## Introduction

The association between sulfur-containing compounds, to which homocysteine (Hcy) and its metabolites cysteine (Cys), glutathione (GSH) and cysteinyl-glycine (Cys-Gly) belong, and origination of some civilization diseases has been well-documented. Among others, it has been recognized that the rise in plasma Hcy levels is a risk predictor of morbidity and mortality of cardiovascular diseases which of the following are accompanied by imbalance in physiological levels of Cys^1–5^. In parallel, the significance of vitamin B6 in human health is well-known^6,7^. Thus, each factor contributing to its depletion in humans might be detrimental to living organisms as the effects of vitamin B6 deficiency are widely known.

Hcy is an intermediate in the metabolic pathway converting methionine to Cys while pyridoxal 5`-phospahte (PLP) is a co-enzyme in a two-step transsulfuration of Hcy to Cys in humans^6–8^. In recent years, it has been reported that aminothiols, in particular Cys easily reacts with PLP in an aqueous environment forming substituted thiazolidine carboxylic acids^9–16^. More importantly, there is evidence that naturally occurring Cys and PLP undergo non-enzymatic condensation producing 2-(3-hydroxy-5-phosphonooxymethyl-2-methyl-4-pyridyl)-1,3-thiazolidine-4-carboxylic acid (HPPTCA)^9–16^, which has been shown to be present in human plasma^16^. However, it is the preliminary data on this matter as the assay has been applied to plasma samples delivered by only thirteen individuals. A rudimentary knowledge of HPPTCA has been gained so far.

The present paper aims to revalidate findings regarding in vivo formation and presence of HPPTCA in humans. Since the provided gas chromatography-mass spectrometry (GC–MS) based assay for assessment of HPPTCA content in human plasma^16^ has been recognized as not powerful enough tool to be used for routine clinical analysis, more effective one is needed to successfully accomplish the study. The liquid chromatography coupled with ultraviolet detection technique (HPLC–UV) was assumed to be suitable for fulfilling the purpose. Important milestones in the validation of the assumption included (1) development of an effective analytical tool based on HPLC–UV for the simultaneous determination of plasma HPPTCA and low molecular-weight thiols, and (2) application of the assay to real samples in order to confirm or exclude the performance of the method as well as verify concentration of plasma HPPTCA reported elsewhere^16^. With a bit of forward thinking, these studies were also essential from the standpoint of providing tool to determine the physiological relevance of the reversible reaction between PLP and Cys.

## Materials and methods

### Reagents and materials

In general, chemicals used in this study were commercially available and at least of analytical reagent grade. Sodium hydrogen phosphate dihydrate, sodium dihydrogen phosphate dihydrate, *tris*(2-carboxyethyl)phosphine (TCEP), HPLC-gradient grade acetonitrile (ACN), trichloroacetic acid (TCA), Cys-Gly, symmetrical disulfides of particular aminothiols, namely homocystine (Hcy_2_), cystine (Cys_2_) and oxidized GSH (Glu_2_) were from Sigma-Aldrich, (St. Louis, MO, USA). Hydrochloric acid, sodium hydroxide and perchloric acid (PCA) were from J.T. Baker (Deventer, The Netherlands). 2-Chloro-1-methylquinolinium tetrafluoroborate (CMQT)^17^ and HPPTCA^16^ were prepared in our laboratory as previously described. Deionized water was produced in our laboratory. Commercially available 50 mg vitamin B6 tablets from Teva Pharmaceuticals (Cracow, Poland), containing active substance pyridoxine hydrochloride, were used.

### Instrumentation

An Agilent 1220 Infinity LC system equipped with a binary pump integrated with two-channel degasser, autosampler, temperature-controlled column compartment and diode-array detector (Agilent Technologies, Waldbronn, Germany) was used for the HPLC–UV experiments. Data acquisition and analysis were performed using an OpenLAB CDS ChemStation software. Analytes were separated on ZORBAX SB-C18 (150 × 4.6 mm, 5.0 µm) column from Agilent Technologies (Waldbronn, Germany). During the study, a Mikro 220R centrifuge with fast cool function (Hettich Zentrifugen, Tuttlingen, Germany), and a FiveEasy F-20 pH-meter (Mettler Toledo, Greifensee, Switzerland) were also used. Water was purified using a Milli-QRG system (Millipore, Vienna, Austria).

### Stock solutions

The stock solution of HPPTCA (1 mmol/L) was prepared daily by dissolving an appropriate amount of HPPTCA powder in deionized water. Then, the solution was kept at 4 °C for no longer than 4 h. The working solutions of HPPTCA were prepared by dilution of standard solution with deionized water as needed and were processed without delay. Importantly, all solutions were protected from light.

The stock solutions of TCEP (0.25 mol/L) and CMQT (0.1 mol/L) were prepared by dissolving an appropriate amount of TCEP or CMQT powder in deionized water. These solutions were prepared freshly as needed.

The stock solutions of 0.1 mol/L Hcy_2_, Cys_2_, Cys-Gly and Glu_2_ were prepared in hydrochloric acid (1 mol/L). These solutions were kept at 4 ˚C for no longer than 7 days without noticeable change of the analyte content. The working solutions of the analytes were prepared daily by dilution of a particular standard solution with 0.2 mol/L phosphate buffer (PBS) pH 7.4 as needed and were processed without delay.

### Biological samples collection

Blood samples (about 2 mL) were collected from individuals after overnight fasting into evacuated tubes containing EDTA by venipuncture, immediately cooled on ice and centrifuged at 5000 × *g* for 15 min at room temperature within 30 min. The separated plasma was delivered to the laboratory in a frozen state using dry ice as a cooling agent. Samples were processed without delay, immediately after defrosting at room temperature, using the procedure described in "Plasma samples preparation" section.

### Plasma samples preparation

Samples were prepared according to the slightly modified method, previously designed to determine levels of total plasma Cys, Hcy, Cys-Gly and GSH^18,19^. The plasma sample (50 μL) was mixed with 50 μL of 0.2 mol/L PBS buffer, pH 7.4, followed by TCEP addition (5 μL, 0.25 mol/L) and put aside for 15 min at room temperature. Thereafter, the mixture was treated with 10 μL of 0.1 mol/L CMQT and left at room temperature for 5 min. Afterwards, 10 μL of 3 mol/L PCA was added. The reaction mixture was incubated at room temperature for 5 h and then kept in a centrifuge at 12,000 × *g* for 10 min at 4 °C. A 10 µL aliquot of the resulting solution was injected onto reversed-phase (RP) column. Each sample was analyzed according to the procedure described in "Plasma samples preparation" section. Note: If the purpose of the analysis is the determination of total content of aminothiols only, samples can be subjected to chromatographic analysis immediately after proteins removal step by means of acidification and centrifugation.

### HPLC–UV conditions

Plasma samples, prepared according to the procedure described in "Plasma samples preparation" section, were analyzed using slightly modified method of R. Głowacki and E. Bald^18,19^. The chromatographic separation was accomplished using ZORBAX SB-C18 (150 × 4.6 mm, 5.0 µm) column with the mobile phase consisted of A) 0.1 mol/L TCA pH 1.7 adjusted with 1 mol/L sodium hydroxide and B) ACN, delivered at the flow rate of 1 mL/min. The chromatographic separation was performed at room temperature using linear gradient elution: 0–8 min 11–40% B; 8–12 min 40–11% B; 12–14 min 11% B. The effluent was monitored with UV detector at 355 nm with the bandwidth of 4 nm using 390 nm ± 20 nm as a reference wavelength.

### Institutional review board statement

The study was conducted according to the guidelines of the Declaration of Helsinki, and approved by the Ethics Committee of the University of Lodz (decision identification code: 7(I)/KBBN-UŁ/II/2020, date of approval 30.03.2020).

### Informed consent statement

Written informed consent was obtained from all subjects involved in the study.

## Results and discussion

Over 10 years of investigations have indicated that the HPLC–UV based method, developed by R. Głowacki and E. Bald^18,19^, provides a useful tool for quantification of total content of Hcy, Cys, Cys-Gly and GSH in various kind of samples, including human plasma^20–23^. Recently, we have recognized that an additional peak of the UV-absorbing 2-*S*-quinolinium derivative of CMQT on the chromatogram appeared, when study samples were left in autosampler for a couple of hours. As a result, experimental work was undertaken to verify the origin of that 5.3 min peak as HPPTCA-delivered signal and then to demonstrate the utility of the method originally dedicated to biologically important aminothiols measurements as well as HPPTCA determination in human plasma. Importantly, sample preparation procedure and chromatographic conditions were not optimized during these studies. Nevertheless, a few experiments were carried out in order to provide reliability of the data from the HPLC–UV assay. All investigations were performed using the procedures described herein. In each case, the appearance of a product peak on the chromatogram and a comparison of its height was used to determine the particular process efficiency. The following (sub)sections of the article provide all necessary information regarding the development, (re)validation, and in-study use of the HPLC–UV based method for the simultaneous determination of HPPTCA and total Hcy, Cys, GSH and Cys-Gly content in human plasma.

### Sample preparation

In the presented study, plasma samples were assayed according to a previously published procedure, designed to determine total Cys, Cys-Gly, Hcy and GSH content, based on HPLC–UV measurements^18,19^. Sample preparation involved disulfides reduction with TCEP, pre-column derivatization with CMQT followed by sample deproteinization via centrifugation preceded by sample acidification with PCA. In general, these steps are typical of such kind of methods^24–27^. At this stage of experiments, considerable attention has been put on examining time course of the reaction between HPPTCA and CMQT. Moreover, each analyte-delivered CMQT-derivative was evaluated in terms of its stability during the method development.

#### Derivatization and stability of CMQT-derivatives

**Figure 1:**
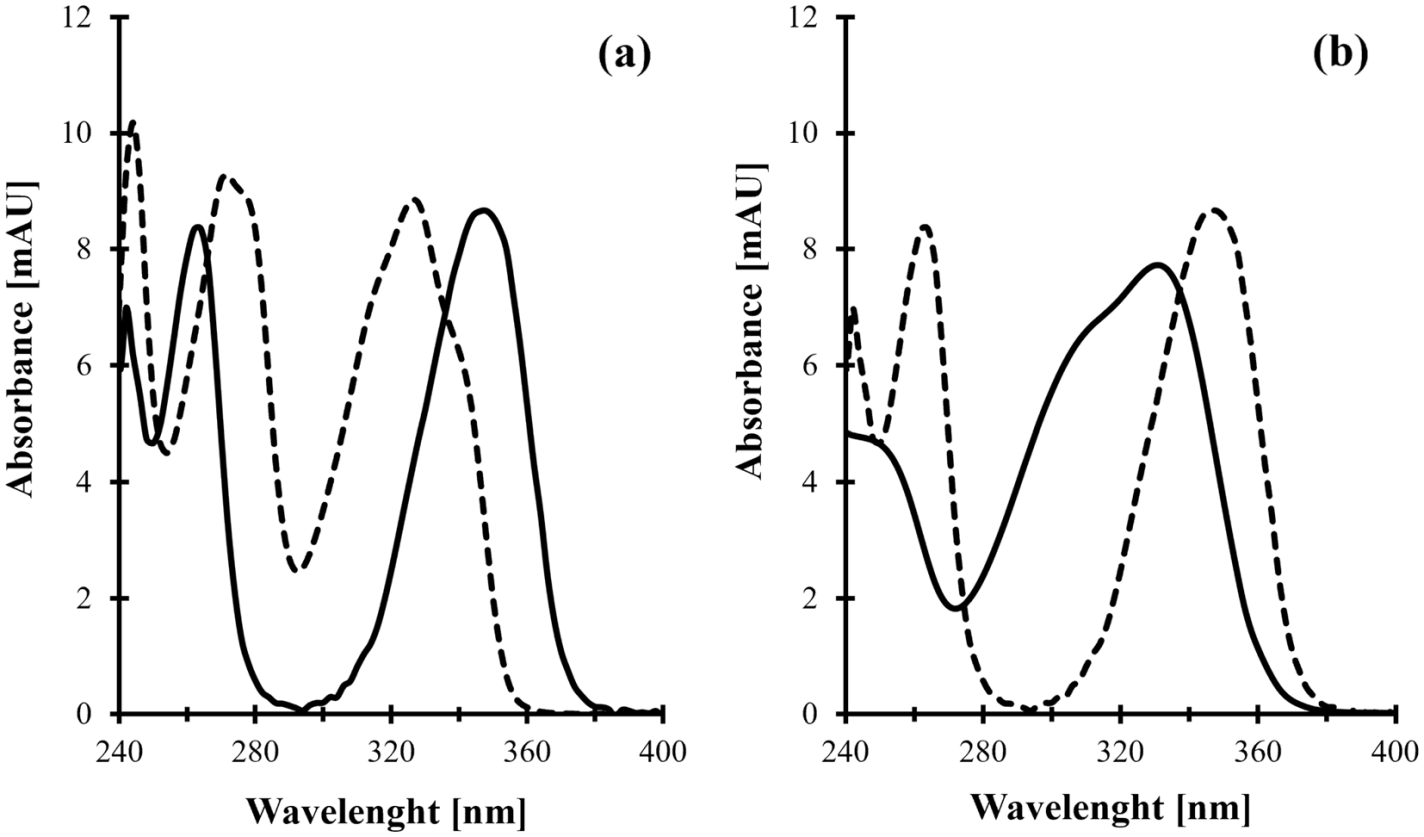
(**a**) Absorption spectrum of HPPTCA-CMQT (solid line) and CMQT (dashed line) obtained by analyzing a standard solution of HPPTCA assayed according to the procedure described in Sects. "Plasma samples preparation" and "HPLC–UV conditions" sections; (**b**) Absorption spectra of HPPTCA (solid line) obtained by analyzing its standard solution assayed according to the procedure described in "HPLC–UV conditions" section and HPPTCA-CMQT (dashed line) obtained as described above.

The presented HPLC–UV assay is based on the derivatization of analytes with the thiol-specific reagent, possessing an active halogen atom as a reactive moiety, namely CMQT. With the regard to HPPTCA, derivatization might be considered not required at first glance since it is a UV-absorbing molecule (Fig. 1). In particular, our earlier experiments have shown that hydrophilic interaction liquid chromatography coupled with beneficial UV properties of HPPTCA enables its direct monitoring^15^. In parallel, it was nonetheless found that the method is not sensitive enough to determine HPPTCA in normal human plasma. Thus, it has been concluded that derivatization would be essential for not only UV detectability enhancement but also extending chemical stability and improvement of chromatographic properties, regarding to HPPTCA.

**Figure 2:**
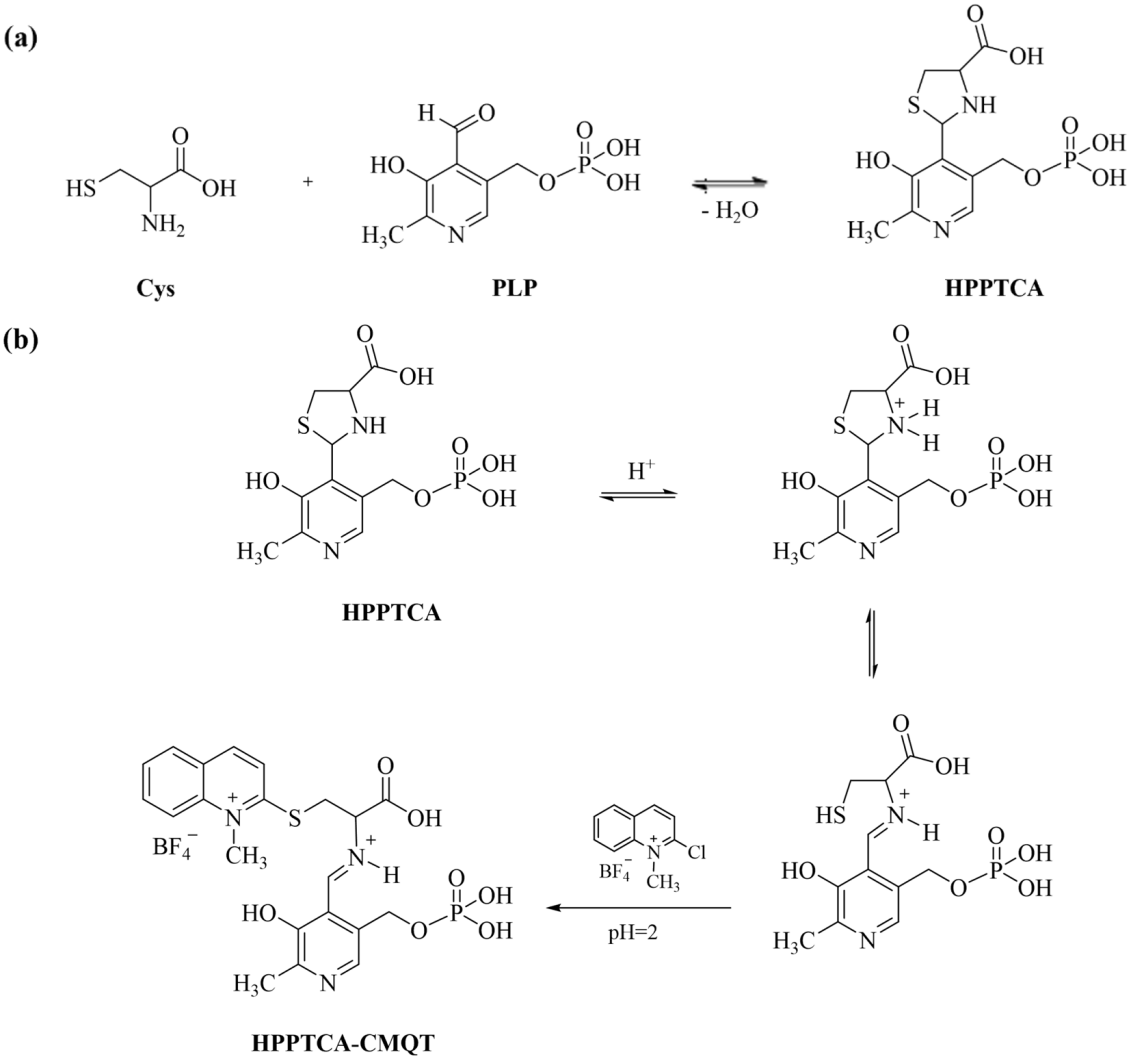
The main research project object. (**a**) The reaction equation of Cys with PLP providing HPPTCA; (**b**) The schematic representation of the reaction involved in the chemical derivatization of HPPTCA with CMQT affording HPPTCA-CMQT derivative.

Thus far, the utility of CMQT as an effective and thiol specific derivatizing agent has been well-documented^25,28–37^. In particular, it has been shown that CMQT can be successfully applied to biological samples (*e.g.* urine, plasma, saliva^25,28–31,33,35,36^, brain homogenates^37^, hen tissues^34^, citrus fruits juices^25^, leaves of cultivated plants^32^, and chemical defined cell media^25^ when determining low- ^25,28–37^ and high-molecular-mass^33^ sulfur-containing compounds by HPLC^25,28,29,32,33,36,37^ and capillary electrophoresis ^25,30,31,34,35^ coupled with UV^25,28,30–37^ or MS^29^ detection. With the exception of thiosulfate^28,35^, it has been demonstrated that CMQT reacts efficiently with low and high molecular-weight sulfur-containing compounds in slightly alkaline aqueous solutions to give corresponding 2-*S*-quinolinium derivatives. During the method development, it has been recognized that derivatization of HPPTCA with CMQT, similarly to thiosulfate, occurs under acidic conditions. Regarding HPPTCA, it has been concluded that it is a complex multi-step process in which the instability of the analyte under acidic and alkaline conditions^14,15^, leading to its hydrolysis, is of key importance. According to the literature data^17,38^, it is very probably that HPPTCA, firstly, undergoes an equilibrium ring opening to the iminium ion intermediate in a hydrogen ion-dependent reaction at acidic pH, resulting in free thiol group release. Then, the reaction relies on the displacement of halide leaving group of CMQT by highly nucleophilic thiol group of analyte, leading to formation of a HPPTCA-CMQT adduct. The possible schematic of the derivatization reaction of HPPTCA with CMQT is depicted in Fig. 2.

Firstly, it was shown that upon derivatization HPPTCA was converted into its corresponding 2-*S*-quinolinium derivative (HPPTCA-CMQT). Analytically advantageous is the fact that reaction with 2-chloroquinolinium salt, namely CMQT is accompanied by bathochromic shift of absorption bands of the derivative compared with its precursors (Fig. 1). Since derivatizing agent was used in a large excess relative to analyte(s), this phenomenon contributed to generation of simpler chromatograms free from interfering peaks. In addition, it has been recognized that derivatization was advantageous from the standpoint of enhancing UV detectability of HPPTCA.

Subsequently, the influence of time on derivatization efficiency was carefully studied. Importantly, time course of the reaction was examined at room temperature in order to simplify the sample pretreatment procedure and improve batch to batch reproducibility. No experiments were undertaken to optimize the temperature of the reaction. In parallel, the stability of the obtained HPPTCA-CMQT and other CMQT-derivatives of interest was evaluated since chemical compounds can be decomposed prior to chromatographic analysis under different circumstances. In particular, this problem has been approached qualitatively and quantitatively in order to observe potential conversion of HPPTCA to Cys and PLP, being its precursors, and to measure of the intactness the analyte(s) in a given matrix under specific use conditions for preselected time intervals, respectively.

**Figure 3:**
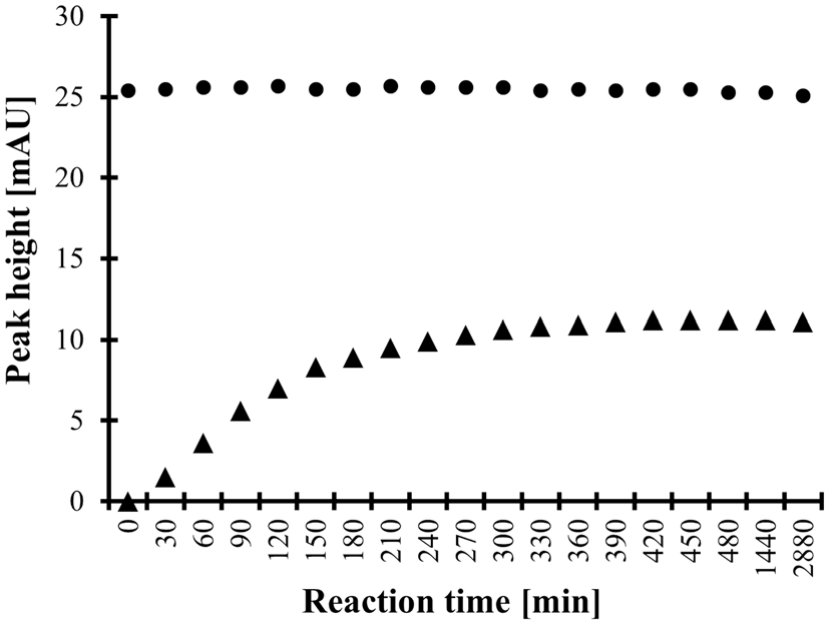
Derivatization reaction yield as a function of time combined with examination of the HPPTCA-CMQT (triangles) and Cys-CMQT (dots) stability in the acidic aqueous solution, expressed as a peak height of the particular derivative. Samples were handled according to the procedure described in "Plasma samples preparation" and "HPLC–UV conditions" section.

In particular, it was established that contrary to low- ^25,28–37^ or even high-molecular-mass^33^ sulfur-containing compounds (reaction time ≤ 30 min), the reaction between HPPTCA and CMQT requires longer reaction time. Notably, it was found that the derivatization reaction is completed in 5 h. As shown in Fig. 3, the increase in peak area of HPPTCA-CMQT was not importantly concomitant with the decrease in peak area of Cys-CMQT. In addition, it has been recognized that HPPTCA-CMQT signal remained stable at least 48 h when samples were left in the temperature not-controlled autosampler. The same phenomenon was observed regarding other CMQT-derivatives of interest as previously reported elsewhere^17,24,39^.

In summary, our research led us to the conclusion that CMQT is suitable derivatizing agent for not only low molecular-weight thiols but also 1,3-thiazolidine-4-carboxylic acid. Importantly, it has been indicated that sample preparation procedure, designed to determine total Cys, Cys-Gly, Hcy and GSH content^18,19^, can be also applied to HPPTCA determination in human plasma. This is a simple one-pot sample processing method in which the overall sample preparation time was estimated to be around 330 min, taking into account all the operations that need to be performed, including disulfides reduction, derivatization, deproteinization, centrifugation, and pipetting, etc. The duration of sample pretreatment procedure was indeed longer in comparison with GC–MS based method enabling plasma HPPTCA determination that requires 140 minutes^16^. Nevertheless, analytically advantageous was an excellent stability of each analyte-delivered CMQT-derivative under experimental conditions allowing to prepare a large batch of samples which contributes to the effort minimization. By contrast with any attempt to measure HPPTCA content using the GC–MS assay^16^, in which samples remain stable no longer than 3 h, there is no necessity of subjecting the test samples to the HPLC–UV analysis just after their preparation in order to produce meaningful results. It nonetheless needs to be emphasized that a successful analysis using the proposed method can only be achieved when the recommended sample handling and management procedures, described herein, are followed. Importantly, the process is accompanied by consumption of 75 µL of inexpensive, although not commercially available, chemicals.

#### Greenness assessment of HPPTCA methods

In recent years, the assessment of an analytical procedure’s greenness is growing in popularity. As a result, several approaches enabling to measure the degree of greenness of analytical methodologies have been developed so far. In the present study, AGREE – Analytical GREEnness metric approach and software (version 0.5 beta)^40–43^, that evaluates analytical procedures considering each of the twelve principles of Green Analytical Chemistry, was employed to assess the greenness of the delivered HPLC–UV method for plasma HPPTCA determination and compare it with reported one^16^. In both cases, equal weights have been importantly set for all twelve principles evaluated.

**Figure 4:**
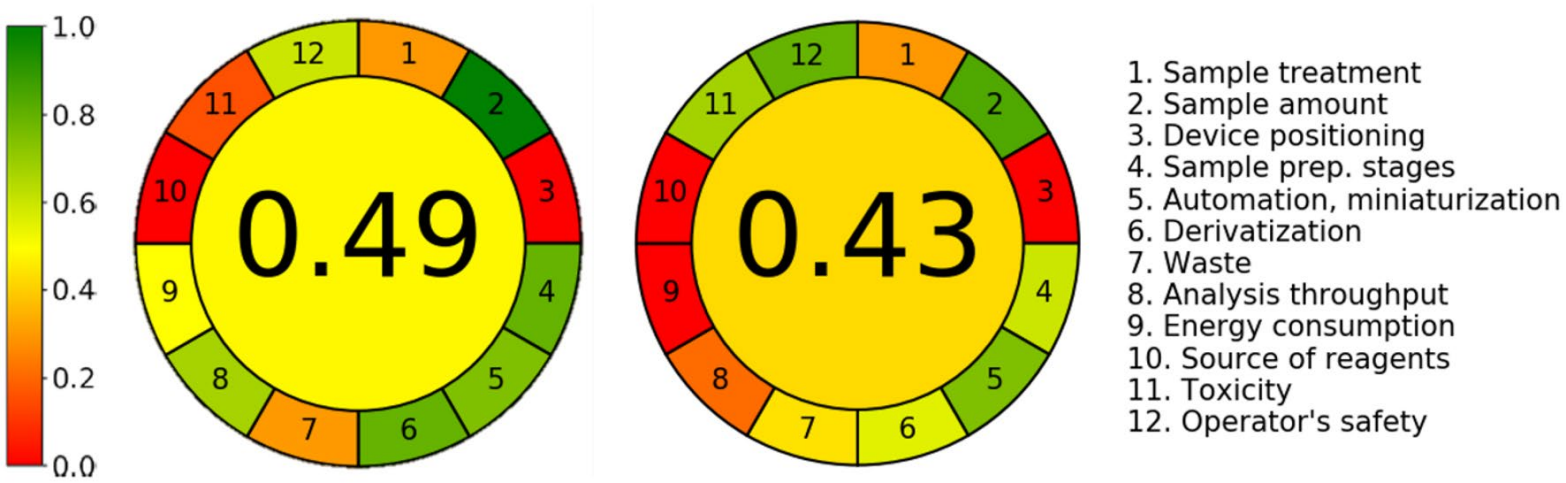
The assessment results with AGREE analysis of procedures for plasma HPPTCA determination by reported GC–MS based method^16^ (right side) and proposed HPLC–UV based method enabling simultaneous determination of HPPTCA and total Cys, Hcy, Cys-Gly, GSH in human plasma (left side).

Regarding to the HPLC–UV assay, the following assumptions have been made in order to assess the analytical procedure’s greenness: the procedure involves an external sample (pre)treatment with a reduced number of steps (principle 1); the volume of plasma is 50 µL (principle 2); the analytical device is positioned off-line (principle 3); the number of distinctive analytical steps is four, including disulfides reduction, derivatization, deproteinization combined with centrifugation, and chromatographic analysis (principle 4); the procedure is semi-automated and involves a miniaturized sample preparation methods (principle 5); derivatization step is required (principle 6); the total amount of waste is 18.625 (g and mL combined), consisting of the sample itself, chemicals and plastic disposable ware used to prepare sample as well as mixture of solvents used during HPLC analysis (principle 7); five analytes are determined in a single run, and the sample throughput is four samples per hour (principle 8); the most demanding technique is HPLC–UV (principle 9); none of the reagents can be obtained from bio-based sources (principle 10), and finally the procedure requires 14 mL of toxic chemical reagents – mobile phase ingredients (principle 11) of which ACN is perceived as highly flammable while TCA toxic to aquatic life (principle 12). In relation to GC–MS assay for analysis of plasma HPPTCA^16^, it has been assumed that the procedure involves an external sample (pre)treatment with a reduced number of steps (principle 1); the volume of plasma is 250 µL (principle 2); the analytical device is positioned off-line (principle 3); the number of distinctive analytical steps is five, including extraction, proteins removal, sample preconcentration by drying under vacuum, derivatization and GC analysis (principle 4); the procedure is semi-automated and involves a miniaturized sample preparation methods (principle 5); derivatization step is required (principle 6); the total amount of waste is 6.5 (g and mL combined), consisting of the sample itself as well as chemicals and plastic disposable ware used to prepare sample (principle 7); one analyte is determined in a single run, and the sample throughput is three samples per hour (principle 8); the most demanding technique is GC–MS (principle 9); none of the reagents can be obtained from bio-based sources (principle 10), and finally the procedure requires 0.25 mL of toxic chemical reagents (principle 11) of which ACN and derivatizing mixture components are perceived as highly flammable (principle 12). The overall AGREE results for the methods under consideration are presented in Fig. 4 as colored pictograms showing the structure of their weak and strong points. As stated by F. Pena-Pereira, et al.^41^, the overall score is shown in the middle of the pictograms while the performance of a particular procedure in each of the assessment criteria is reflected with the red-yellow-green color scale. In addition, values close to one and dark green color indicate that the assessed procedure is greener.

In particular, using AGREE – Analytical GREEnness metric approach we were able to conclude that the presented herein HPLC–UV assay (score 0.49) is comparable in terms of greenness to the GC–MS based method designed to determine HPPTCA in human plasma (score 0.43)^16^. However, the distribution of weak and strong points differs in both cases (Fig. 4). Finally, it has been assumed that the proposed HPLC–UV based method for determination of HPPTCA and total Cys, Hcy, Cys-Gly, GSH in human plasma can be considered environmentally-friendly thanks to the possibility of carrying out the chemical analysis on a very small scale combined with low consumption of hazardous chemicals and laboratory disposable plastic ware at the stage of sample processing, its high-throughput potential and simplicity in sample preparation (Fig. 4).

### Chromatographic and detection conditions

**Figure 5:**
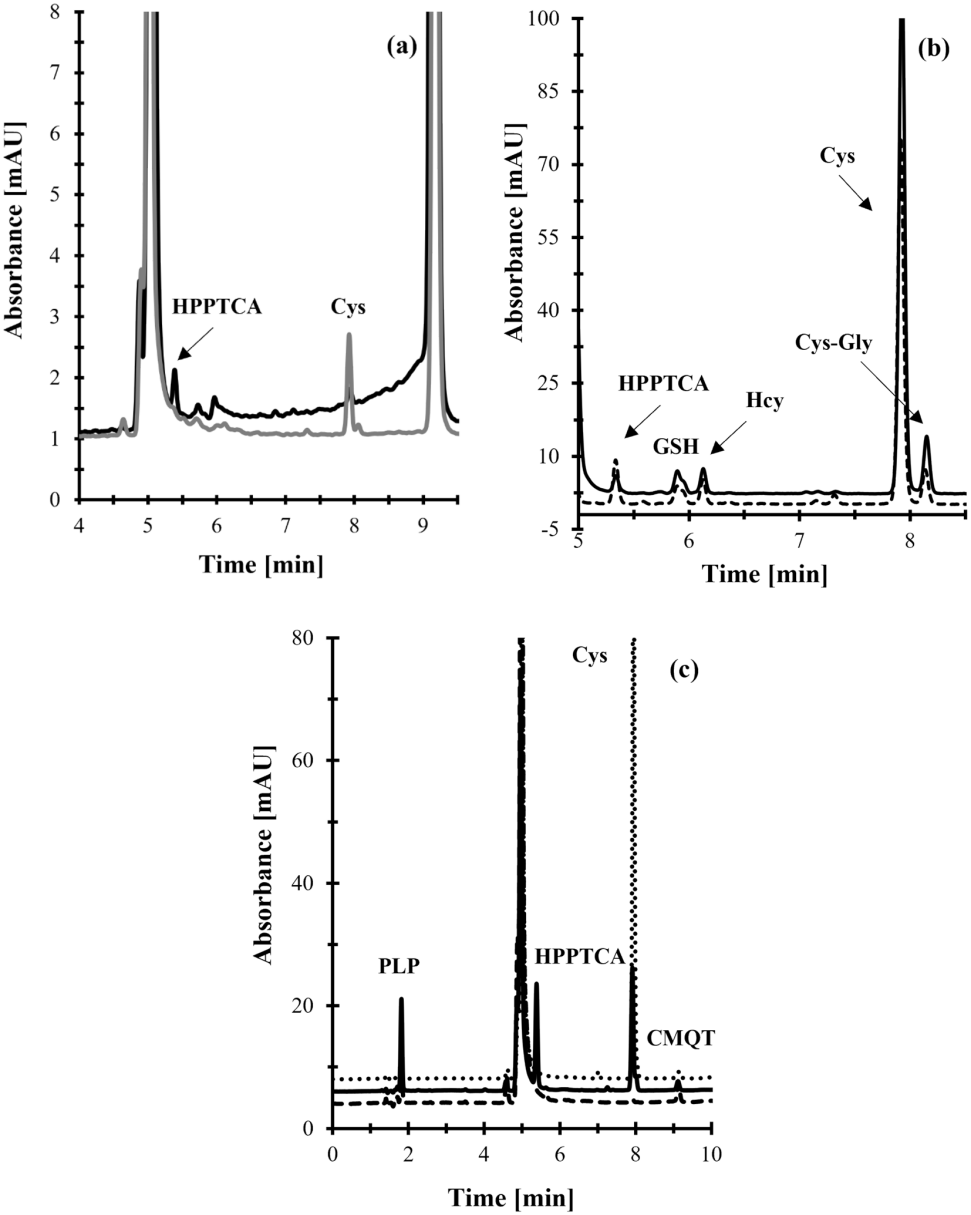
A representative chromatograms of standard solutions and human plasma prepared according to the procedure described in "Plasma samples preparation" section. Chromatographic conditions were as described in "HPLC–UV conditions" section. (**a**) Deproteinized human plasma sample (black line) and the same sample treated with sodium hydroxide (grey line); (**b**) Normal human plasma collected before (solid line) and after oral ingestion of vitamin B6 (dashed line); (**c**) Standard solution of HPPTCA (black line), Cys / Cys_2_ (dotted line) and PLP (dashed line).

Numerous methods, predominantly based on liquid phase separation techniques, for Cys, Hcy, Cys-Gly and GSH determination have been developed thus far^24–27^. Undoubtedly, these techniques are tools of first choice for the determination of low molecular weight hydrophilic compounds. Interestingly, none of them allows the simultaneous determination of the above-mentioned thiols and HPPTCA in human plasma. To the best of our knowledge, only one assay based on GC–MS technique, applied for identification and quantification of HPPTCA in human plasma has been elaborated so far^16^. Unfortunately, this approach is not free from restrictions, indicating the need for providing more effective analytical tool. Since aminothiols and HPPTCA exhibit high compatibility with liquid phase separation techniques, particularly in relation to their good solubility in commonly used mobile phases, we have decided to demonstrate its utility for the present purposes. To successfully complete the project, plasma samples were assayed according to previously published by R. Głowacki and E. Bald, the ion pair RP-HPLC–UV based method^18,19^. Importantly, it has been recognized that utility of the assay originally dedicated to Cys, Hcy, Cys-Gly and GSH, can be extended to HPPTCA determination in human plasma. As all analytes were well-resolved under the conditions described in the literature^18,19^, no further experimental work was undertaken to optimize separation and detection conditions during new method development. The representative chromatograms are shown in Fig. 5.

Under the conditions described in "HPLC–UV conditions" section, all analytes eluted within 14 min and their corresponding peaks were easy to distinguish from the responses of all the concomitant matrix components. Equally importantly, each time the elution profile of blank samples was free from any interference at the retention time of the analytes (Fig. 5). The order of elution was as follows: HPPTCA (5.3 min), GSH (5.9 min), Hcy (6.1 min), Cys (7.9 min) and Cys-Gly (8.1 min), being in agreement with predicted increasing hydrophobicity of their CMQT-derivatives.

The identification and confirmation of the target compounds, in particular HPPTCA were performed by analyzing the standard solution of analytes processed according to the procedure described in "Plasma samples preparation" and "HPLC–UV conditions" section. Each solution of sulfur-containing compound was prepared separately and then was processed in order to ascertain that a single analyte did not yield more than one chromatographic peak. The analyses were carried out by HPLC–UV system which was set to collect both time information as well as spectral information. Importantly, it has been recognized that HPPTCA-CMQT derivative, similarly to other CMQT-derivatives of interest, exhibits a well-defined absorption peak near 355 nm (Fig. 1) indicating that all analytes can be monitored at given wavelength of 355 nm.

Additional experiments were performed to confirm the origin of 5.3 min peak. As stated before, the condensation reactions between aldehydes and Cys to 1,3-thiazolidine-4-carboxylic acids are reversible at acidic as well as alkaline pH^14–16^. Thus, the same blank plasma samples were assayed, according to the procedures described herein ("Plasma samples preparation" and "HPLC–UV conditions" section), before and after treatment with sodium hydroxide (1 mol/L). Importantly, plasma samples were deproteinized by ultrafiltration over 10 kDa cut-off membranes to remove protein-bound Cys, representing almost 65% of plasma Cys^1,25,27^, to increase sensitivity of these assays. In particular, typical HPPTCA-delivered peak disappearance with concomitant increase in the Cys peak have occurred when plasma samples were treated with sodium hydroxide (Fig. 5). In parallel, it was found that base hydrolysis converts plasma HPPTCA to PLP. To this end, the base sensitivity and the generation of Cys and PLP after base hydrolysis confirmed the identity of the 5.3 min peak as derived from HPPTCA present in human plasma.

Finally, the confirmation of the origin of each analyte-delivered peak and quantification of the compound of interest in real samples were based upon comparison of retention time and absorption spectra with the corresponding set of data obtained by analyzing an authentic compound.

### Validation of the method

The validity of the method for the determination of total Cys, Hcy, Cys-Gly and GSH content in human plasma has been demonstrated earlier^18,19^. Thus, full validation of the HPLC–UV assay was performed in terms of HPPTCA. In general, the same approach was used for this purpose as was reported in our earlier paper concerning GC–MS assay for analysis of plasma HPPTCA^16^. The selection of elements and acceptance criteria of the method development and validation was made based on the United States Food and Drug Administration guidance for bioanalytical methods validation^44^. Particularly, selectivity, linearity, the limit of quantification (LOQ), accuracy, and precision were evaluated. In addition, the matrix and carry-over effects were evaluated during method validation. Some parameters which can be checked among system suitability testing were assessed. Detailed data regarding all evaluated validation parameters are presented herein.

#### System suitability

System suitability parameters such as repeatability of chromatographic retention, expressed as the coefficient of variation (CV) of retention time, asymmetry factor, and number of theoretical plates were selected during method validation to determine instrument performance under optimized conditions. The system suitability tests were assessed by replicate measurements of the calibration standards at the upper LOQ (ULOQ) as a part of a linearity assessment. Importantly, good system suitability was shown, ensuring that the system was performing in a manner that leads to the production of accurate and reproducible data. The CV value of retention time was 0.21% (acceptance criteria ≤ 1%), the mean asymmetry factor was 1.12 (acceptance criteria 0.8–1.5), and number of theoretical plates was 40,355 (acceptance criteria ≥ 2000).

#### Linearity

External standard calibration method was used to assess the calibration range of the method. For this purpose, multilevel calibration curves were generated and run in triplicate over 3 subsequent working days. The calibration curves consisted of a blank sample and six calibrators in the range of 1–100 µmol/L in plasma, including LOQ. Calibration standards were prepared in laboratory-made pooled plasma by spiking the matrix with known quantities of HPPTCA which concentrations were chosen on the basis of the concentration range expected in study samples^16^. Pooled plasma made up of small pools of the specimens from all donations was produced in our laboratory. Since plasma samples free of HPPTCA were not available, the endogenous concentration of the analyte was evaluated before the calibration curve preparation by triplicate analysis. The linearity was evaluated graphically by visually inspecting a plot of the peak height as a function of the analyte concentration as well as using the least-squares regression model to describe the concentration–response relationship. In particular, curves’ correlation coefficient (R) was monitored showing that the instrument response was directly proportional to the analytes’ concentration within the intended quantitation range (Table 1). Conducted experiments also indicated that only the signal peak height of HPPTCA-CMQT derivative increased linearly with the growing concentration of the analyte. Importantly, substantial changes in the slope of particular regression line obtained across the day as well as over 3 subsequent days were not observed (Table 1). It has been nonetheless encountered that the analytical method might have been affected by matrix components. Thus, matrix effect was investigated during the validation and implementation of the method.

**Table 1:** Validation data corresponding to intra-assay measurements (n = 3).

Regression equation	R	CV slope (%)	Linear range (µmol/L)	Intra-assay precision (%)		Intra-assay accuracy (%)		LOQ (µmol/L)
				Min	Max	Min	Max	
y = 0.2820x + 17.38	0.9995	1.89	1–100	2.48	6.99	92.74	105.57	1

CV, coefficient of variation; LOQ, limit of quantification; R, correlation coefficient.

#### Accuracy and precision

Accuracy and precision of the assay, referring to intra- and inter-day measurements, were evaluated as a part of a linearity assessment. The precision and accuracy were expressed as CV of measurement repeatability and the percentage of analyte recovery, respectively. In particular, accuracy was calculated by expressing the mean measured amount as a percentage of added amount of HPPTCA with the use of the following formula *Accuracy (%)* = *[(measured amount* × *endogenous content) / added amount]* × *100*. Intra-assay precision and accuracy were demonstrated by triplicate analysis of freshly prepared calibration standards, which referred to pooled plasma samples containing known amounts of the analyte at three different levels, including one close to the lower LOQ (LLOQ), one in the middle of the quantitation range, and one at ULOQ and equaled to 10, 50 and 100 µmol/L in plasma, respectively. Experiments concerning the estimation of intermediate accuracy and precision were repeated, in the same manner, over 3 subsequent days. All concentrations were tested with the use of calibration curves prepared specifically on that occasion. Importantly, obtained results from analytical runs met the acceptance criteria. The accuracy ranged from 92.74 to 105.57% and 95.43 to 115.73% for intra- and inter-day variation, respectively. The precision did not exceed 6.99% of CV at any examined concentration level. It varied from 2.48 to 6.99% and 0.84 to 6.98% for intra- and inter-day measurements, respectively.

#### Matrix effect

The matrix effect between different independent sources, defined as an alteration in analyte(s) response due to the presence of interfering and usually unidentified sample components, was evaluated in a relevant volunteers population. Apart from selectivity studies, the matrix effect evaluation involved comparing calibration curves of the six individual sources of plasma samples against a calibration curve of the pooled matrix. Importantly, it was recognized that the slope of the regression lines did not deviate by more than 5.49% denoting the absence of any matrix effect. In fact, with this difference in slope, there only would be a few percent errors in analytical results using any of the regression lines to quantify the sample. Thus, traditional calibration curve approach was used to establish the levels of the analytes in plasma samples as it provides the procedure’s reliability along with the effort minimization.

#### Carry-over

Carry-over between samples, meaning the appearance of an analyte in a sample from a preceding sample, can occur in analytical methods. Since it may have an impact on the accuracy and precision of the study sample concentrations, the potential of carry-over was thus investigated in the present study as a part of the linearity evaluation. In order to assess the carry-over, blank standard solution sample(s) were placed after the calibration standard at the ULOQ. Importantly, each time the response of blank samples was as high as the background signal indicating that carry-over was effectively eliminated during method development.

#### Selectivity

In relation to HPPTCA, the selectivity of the analyte in the presence of any other UV-absorbing sample components was verified at first attempt during studies concerning the identification and confirmation of the origin of HPPTCA-delivered peak as described in section 3.2. In addition, selectivity studies assessed interferences originating from matrix components being precursors of HPPTCA formation. They included Cys, Cys_2_ and PLP, which are present in plasma specimens^1,6,7,25,27^. The blank standard solution and standard solution of each of them were assayed according to the procedure described in "Plasma samples preparation" and "HPLC–UV conditions" section. As shown in Fig. 5, the elution profile is free from any interferences at the retention time of HPPTCA-CMQT peak. Under these conditions, the PLP does not react with CMQT and its corresponding peak is eluted before the column dead volume, while Cys and Cys_2_ gives a Cys-CMQT derivative eluting at 7.9 min. Afterwards, normal plasma samples from six individual sources and the same samples spiked with HPPTCA precursors were assayed without delay according to the procedures described herein ("Plasma samples preparation" and "HPLC–UV conditions" section). No increase in the peak area / height of HPPTCA was observed. Moreover, selectivity studies encompassed the evaluation of peak purity. The same spectra, acquired in different sections of a HPPTCA-CMQT peak, were importantly observed indicating its purity.

#### The limit of quantification

LOQ evaluation was done as a part of the intra-assay precision and accuracy assessment for the calibration range. The LLOQ, which equals to 1 µmol/L in plasma for HPPTCA, was accepted as LOQ. Importantly, this concentration of the analyte produced easy to distinguish from the background noise and reproducible detector response with a precision that did not exceeded 3.06% and accuracy ranged from 93.05 to 104.56%. In addition, the estimated LOQ value closely corresponded to the LOQ determined experimentally by signal-to-noise method. In this method, a surrogate matrix (0.9% sodium chloride in 0.1 mol/L PBS pH 7.4) was enriched with decreasing concentrations of the analyte and handled according to the procedures described in "Plasma samples preparation" and "HPLC–UV conditions" sections until the injected amount of HPPTCA resulted in a peak nine-times as high as the baseline signal. Since it was difficult to obtain a blank plasma, a surrogate matrix was used. The obtained LOQ value was similar to that published earlier concerning determination of HPPTCA in human plasma by GC-MS^16^.

In conclusion, it has been demonstrated among method validation that the HPLC–UV assay is suited for the analysis of plasma samples in terms of HPPTCA as well as some low molecular-weight thiols. Regarding HPPTCA, it has been particularly shown that the method is sensitive enough and has suitable levels of precision, accuracy and linearity, falling within acceptable tolerance limits^44^. Importantly, it has been recognized throughout the application of the method that carry-over between samples did not occur in analytical method and matrix has a negligible impact on the assay results. Based on the analysis of validation data, it has been concluded that the performance of the presented herein HPLC–UV based method is generally comparable to previously published procedure, designed to determine plasma HPPTCA content, based on GC–MS measurements^16^.

### Application of the method

The validated HPLC–UV assay was applied to plasma samples in order to prove its utility. A group of eighteen apparently healthy anonymous individuals, belonging to an ethnically homogeneous group, was involved in the study. They were not supplemented with the analytes, neither HPPTCA precursors (Cys, Cys_2_ and PLP) before sample collection. Since it has been recognized that the assay is not affected by matrix components, an external standard addition method was used to establish plasma levels of the analytes in study samples handled according to the procedures described in Sects. Plasma samples preparation and "HPLC–UV conditions" section. Importantly, the compounds of interest were detected in all study samples. The estimated concentrations of plasma Cys, Hcy, GSH, Cys-Gly and HPPTCA, based on data obtained by triplicate analysis of a particular sample from an individual source, were within the range typical for healthy people and varied from 171.5 to 296.4 µmol/L, from 5.2 to 13.2 µmol/L, from 1.8 to 7.3 µmol/L, from 19.0 to 41.8 µmol/L, from 19.2 to 65.6 µmol/L, respectively. Importantly, these values were similar to those previously reported, using different assays, indicating the reliability of our method^16,18,19,26,27,29,31,33,36,39^. Moreover, it has been recognized that the concentration of plasma HPPTCA is about fivefold higher than the concentration of plasma reduced Cys^1,25^ as well as about 1000-fold higher than plasma PLP^6–8^, in agreement with previous studies performed using the GC–MS assay^16^. Moreover, using least squares regression model to evaluate the association between HPPTCA level and total Cys content in human plasma, we have found that there was a moderate positive correlation (R equals to 0.63) between these two variables. It is therefore reasonable to assume that plasma HPPTCA content depends on concentration of Cys and PLP, among other things. Nevertheless, it should be clearly stated that this supposition should be carefully reinvestigated as a relatively small group of people has participated in the experiment.

In addition, the HPLC–UV assay was used to reevaluate the reactivity of Cys and PLP in vivo as this method makes possible to simultaneously monitor both the HPPTCA and Cys in study samples. The experiment was conducted according to the research protocol previously described in the literature^16^. Two apparently healthy adult volunteers were involved in the experiment. One dose of vitamin B6 (200 mg) in the form of dietary supplement, which did not exceed the recommended dose per day, was administered orally to them. Blood samples were collected just before and after 12 h ingestion of vitamin B6-containg pharmaceutical preparation, as described in "Biological samples collection"  section, and then handled according to procedure described in "Plasma samples preparation" and "HPLC–UV conditions" sections. This in vivo experiments have confirmed the identity of the peak eluting at 5.3 min indicating that it is derived from HPPTCA present in human plasma. Notably, we found that Cys-CMQT peak decreased slightly in parallel with a marked increase in the HPPTCA-derived peak after plasma donors ingested orally vitamin B6 (Fig. 5), and the same observation was made by J. Piechocka, et al^16^. Interestingly, no change in the Hcy-CMQT peak was noticed.

## Final remarks

A new (first) method for simultaneous assessment of HPPTCA as well as total Hcy, Cys, GSH and Cys-Gly content in human plasma has been provided. The assay is primarily characterized by a streamlined sample preparation procedure involving disulfides reduction, derivatization and sample deproteinization followed by HPLC–UV analysis. Analytically advantageous is an excellent stability of the obtained UV-absorbing 2-*S*-quinolinium derivatives under experimental conditions which allows to prepare multiple samples in a single analytical run. Moreover, this report provides evidence that the practical application of CMQT in sulfur-containing compounds analysis can be extended to 1,3-thiazolidine-4-carboxylic acids.

Even more importantly, the HPLC–UV assay provides a new useful tool that has the potential for facilitating studies of the role of the above-mentioned compounds in health and disease, in particular HPPTCA. Significance of HPPTCA to living organisms still remains substantially unknown. Interestingly, these experiments have produced compelling evidence that vitamin B6 supplementation elevates plasma HPPTCA which may be accompanied by reduction in plasma Cys or even toxic Hcy levels. This theory may provide an explanation for the beneficial impact of vitamin B6 supplementation. More importantly, present studies conducted with the use of eighteen samples have supported the conclusion that HPPTCA is the most abundant component of vitamin B6 pool in human plasma^16^. In particular, the facile formation of HPPTCA as well as its possible decomposition leading to Cys and PLP release, point to a conclusion that PLP is stored in the human body in the form of HPPTCA. However, the research using larger control and experimental groups is needed to obtain compelling evidence supporting the conclusion. In addition, it would be reasonable to monitor the concentration of all known forms of vitamin B6 in humans concomitantly with HPPTCA. Hopefully, the produced results will feature in further research in the field, in particular involving biostatistical analysis of medical data in near future.

## Data Availability

Data is contained within the article. In addition, the dataset generated and analyzed during this study, which contributed to the article, can be made available by the corresponding authors (J.P. and R.G.) upon reasonable request as long as the request does not compromise intellectual property interests.
